# The Effect of Age, Sex, Area Deprivation, and Living Arrangements on Total Knee Replacement Outcomes: A Study Involving the United Kingdom National Joint Registry Dataset

**DOI:** 10.2106/JBJS.OA.17.00042

**Published:** 2018-04-24

**Authors:** Hannah B. Edwards, Michèle Smith, Emily Herrett, Alexander MacGregor, Ashley Blom, Yoav Ben-Shlomo

**Affiliations:** 1 University of Bristol, Bristol, United Kingdom; 2 NIHR Collaboration for Leadership in Applied Health Research and Care West, Bristol, United Kingdom; 3 London School of Hygiene and Tropical Medicine, London, United Kingdom; 4 University of East Anglia, Norwich, United Kingdom; 5 North Bristol National Health Service Trust, Bristol, United Kingdom

## Abstract

**Background:**

Total knee replacement (TKR) is a common procedure for the treatment of osteoarthritis that provides a substantial reduction of knee pain and improved function in most patients. We investigated whether sociodemographic factors could explain variations in the benefit resulting from TKR.

**Methods:**

Data were collected from 3 sources: the National Joint Registry for England, Wales, Northern Ireland, and the Isle of Man; National Health Service (NHS) England Patient Reported Outcome Measures; and Hospital Episode Statistics. These 3 sources were linked for analysis. Pain and function of the knee were measured with use of the Oxford Knee Score (OKS). The risk factors of interest were age group, sex, deprivation, and social support. The outcomes of interest were sociodemographic differences in preoperative scores, 6-month postoperative scores, and change in scores.

**Results:**

Ninety-one thousand nine hundred and thirty-six adults underwent primary TKR for the treatment of osteoarthritis in an NHS England unit from 2009 to 2012. Sixty-six thousand seven hundred and sixty-nine of those patients had complete knee score data and were included in the analyses for the present study. The preoperative knee scores were worst in female patients, younger patients, and patients from deprived areas. At 6 months postoperatively, the mean knee score had improved by 15.2 points. There were small sociodemographic differences in the benefit of surgery, with greater area deprivation (−0.71 per quintile of increase in deprivation; 95% confidence interval [CI], −0.76 to −0.66; p < 0.001) and younger age group (−3.51 for ≤50 years compared with 66 to 75 years; 95% CI, −4.00 to −3.02; p < 0.001) associated with less benefit. Cumulatively, sociodemographic factors explained <1% of the total variability in improvement.

**Conclusions:**

Sociodemographic factors have a small influence on the benefit resulting from TKR. However, as they are associated with the clinical threshold at which the procedure is performed, they do affect the eventual outcomes of TKR.

**Level of Evidence:**

Prognostic Level IV. See Instructions for Authors for a complete description of evidence.

Total knee replacement (TKR) is a common surgical treatment for knee osteoarthritis^1,2^. The procedure reduces symptoms in the majority of patients^3-6^ and is associated with low rates of complications and mortality^7-11^. However, 10% to 30% of patients experience persistent pain and disability following surgery^12-15^.

Sociodemographic factors are associated with inequalities in mortality, morbidity, and functional status^16-19^ and may help to explain the variation in outcomes following TKR. Evidence on the effect of sociodemographic factors on the outcomes of TKR is conflicting. Previous studies have demonstrated either no difference in outcomes related to sex^20-22^ or worse outcomes in female patients^23-26^. Both older^26-29^ and younger^30,31^ age have been associated with worse outcomes, and some studies have demonstrated either no effect or a U-shaped effect of age^21,22,25^. A U-shaped effect is when people at both ends of the spectrum (e.g., the youngest and the oldest) both have a worse outcome compared to people in the middle of the spectrum (e.g., those close to the mean age). Deprivation^5,22,24,27,29,32^, living alone, lower social support, and being unmarried have also been linked to worse outcomes^22,29,33,34^. These findings need to be interpreted cautiously because of methodological limitations such as retrospective reporting of outcomes, failure to adjust for surgical and clinical confounding factors, and the limited statistical power from small sample sizes.

The exploration of associations between sociodemographic factors and clinical outcomes is important because these results may be used to advise patients preoperatively and should be considered when comparing surgeon and organizational variability in performance. An association, if causal, might also suggest possible mechanisms that could lead to more appropriate interventions. We aimed to elucidate the role of sociodemographic factors in the outcome of TKR in England with use of prospective, population-based data from the largest national joint registry in the world.

## Materials and Methods

### Data Sources

Data were collected from 3 sources: the National Joint Registry for England, Wales, Northern Ireland, and the Isle of Man (NJR)^35^; National Health Service (NHS) England Patient Reported Outcome Measures (PROMs)^36^; and NHS England Hospital Episode Statistics (HES)^37^.

The NJR collects data at the time of the procedure for all patients who undergo TKR in England, Wales, Northern Ireland, and the Isle of Man. These data include age, sex, type of knee constraint, and method of knee fixation. Patients with partial, unicondylar, and patellofemoral replacements were excluded from the present study, as were those who underwent revision procedures and procedures for any reason other than osteoarthritis.

HES data are collected routinely by health-care staff at the point of care. The current analysis was restricted to patients who were recorded as Caucasian because the numbers in the ethnic minority groups were small and were unlikely to have sufficient power to detect differences in outcomes. Postal codes were used to derive area deprivation scores according to the English Indices of Multiple Deprivation^38^, a multi-domain, census-based ecological indicator of individual socioeconomic status. Small areas with approximately 1,500 residents were then ranked from least to most deprived with use of a weighted score derived from census data covering 7 domains (income, employment, education, health, crime, barriers to housing, and services and living environment). These area scores were categorized into quintiles, with quintile 1 representing the least deprived 20% of patients and quintile 5 representing the most deprived 20%.

National PROMs have been routinely collected by NHS England since 2009 with use of questionnaires that are sent to patients 2 weeks before and 6 months after the procedure. These questionnaires include data on comorbidities, duration of knee symptoms, postoperative complications, and the Oxford Knee Score (OKS) assessment (see Appendix). The OKS measures patient-reported knee pain and function over the preceding 4 weeks, with the score being categorized as poor (0 to 27), fair (28 to 33), good (34 to 41), or excellent (42 to 48)^39^. The OKS is widely used and validated^39-41^, and previous studies have demonstrated that the outcome at 6 months postoperatively is a good predictor of the longer-term outcome at 2 to 8 years postoperatively^3,28,42^. Data linkage among the 3 datasets is described in the NJR annual report^35^.

### Sample Size

**Fig. 1:**
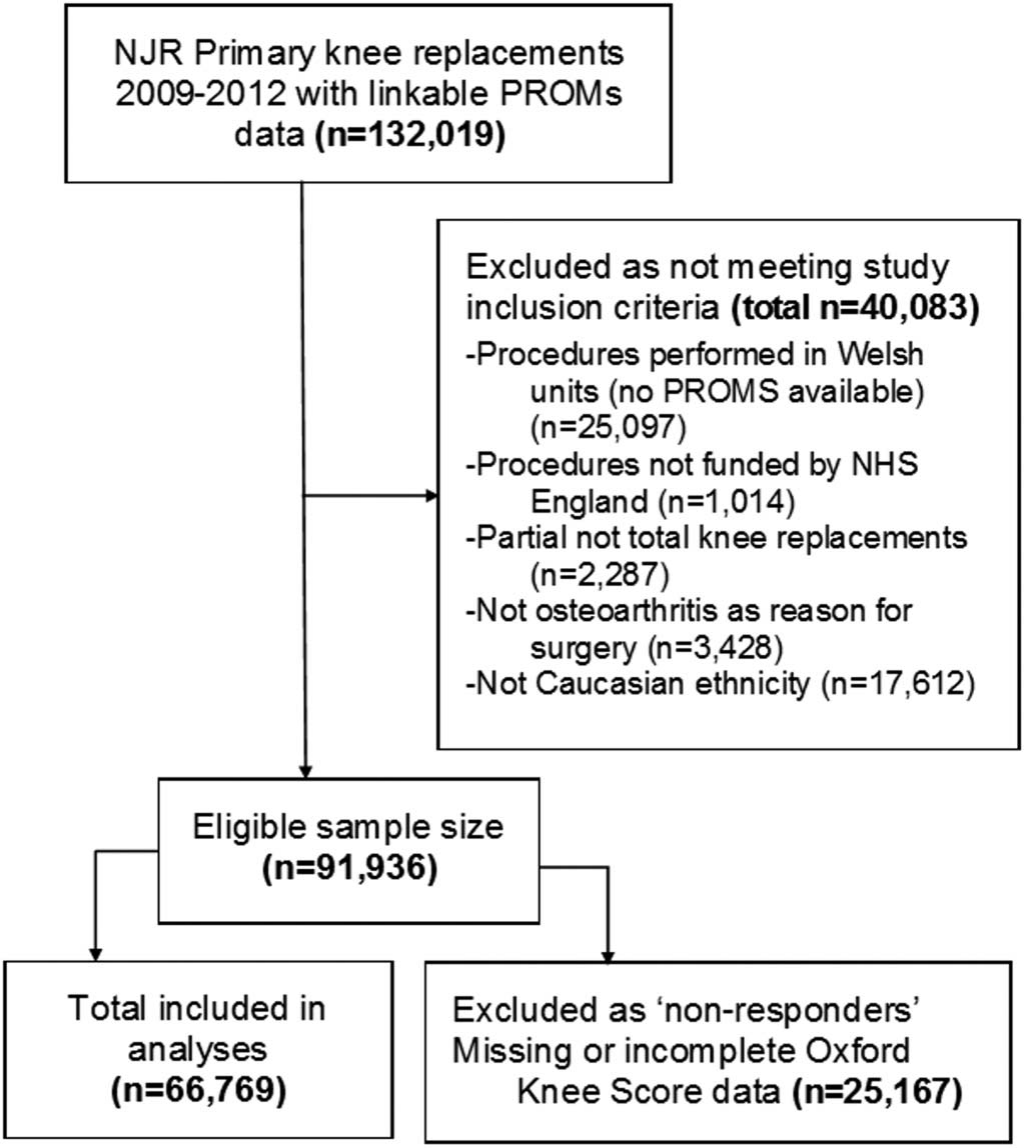
Flow diagram showing the inclusion and exclusions of records at each stage of the study.

Ninety-one thousand nine hundred and thirty-six Caucasian adults underwent primary TKR for osteoarthritis in an NHS England unit from April 1, 2009, to December 31, 2012. Sixty-six thousand seven hundred and sixty-nine of those patients had complete OKS data and were included in the analyses for this study (Fig. 1). Non-responders to the postoperative questionnaires were compared with responders to assess sampling bias and generalizability. Given the large sample size, there was >99% power to detect a difference of 5% in the proportions of patients who did not experience an improvement of ≥3 points in the knee score (i.e., the minimum clinically important difference^39,43^) at the 0.05 significance level.

### Ethical Approvals

This work fell within the remit of the Bristol Musculoskeletal Research team’s permissions to work on NJR, PROMs, and HES data. All original data were collected with patient consent. Only anonymized data were used. The study was also approved by the London School of Hygiene and Tropical Medicine Ethics Committee for an MSc project.

### Statistical Methods

We examined sociodemographic differences in (1) mean preoperative knee scores, to determine if sociodemographic differences existed prior to surgery; (2) mean 6-month postoperative knee scores, to determine if outcomes differed following surgery; and (3) mean change in knee scores (calculated as the postoperative score minus the preoperative score), to determine whether sociodemographic factors influenced improvement following surgery. Our null hypotheses were that there would be no sociodemographic differences in mean preoperative scores, postoperative scores, or preoperative-to-postoperative change in scores.

Age groups were modeled as a categorical variable (≤50 years, 51 to 65 years, 66 to 75 years, 76 to 85 years, and ≥86 years), so we did not assume a linear association. The English Indices of Multiple Deprivation score was tested as a categorical variable, but because the patterns looked linear, the analysis was run as an ordinal variable; as a result, the coefficients are for a 1-quintile increase in deprivation. Other variables that were adjusted for in the analysis were coded as binary.

Linear regression modeling was utilized to estimate the association between sociodemographic factors and knee scores (with the influence of each variable being expressed as beta coefficient, 95% confidence interval [CI], p value), and R^2^ statistics were utilized to identify how much of the total variability of outcome could be explained by sociodemographic factors. The interactions between age and sex and between age and deprivation were investigated. Regression diagnostics were performed to check for normality of residuals.

We adjusted our multivariable models to control for potential confounding as follows. In the preoperative model, we adjusted for sociodemographic factors (age, sex, area deprivation, and living arrangements), symptom duration, and comorbidities. In the postoperative and change-score models, we adjusted for these same sociodemographic factors and additionally adjusted for postoperative complications, knee implant type, and knee constraint type. In the change-score model, we adjusted for these same sociodemographic factors and additionally adjusted for preoperative score as this variable was expected to constrain the change in score as a result of the “ceiling effect” of the scoring system. The “ceiling effect” is that patients with lowest preoperative scores have the most room for improvement and therefore would be expected to show the greatest change in score^39^. The adjusted models included only the records that had complete data for all of the included variables, which resulted in a small reduction in sample size (from 66,769 to 62,941 observations) and made very little difference to the unadjusted model estimates.

We also ran a sensitivity analysis that included body mass index (BMI) to see if this altered the associations. BMI was not included in the main model because data were missing for approximately 30% of the patients, thereby reducing precision and, potentially, generalizability.

## Results

### Sample Characteristics

The 66,769 patients who responded to both OKS questionnaires were comparable with the 25,167 patients who did not. Non-responders more frequently came from deprived areas and may have been slightly worse off clinically, with fractionally lower preoperative knee scores, longer duration of symptoms, and more comorbidities. Other demographic differences, although statistically significant because of the large sample size, were of very small magnitude (e.g., the proportion of female patients was 56.5% among responders, compared with 57.8% among non-responders) (Table I).

**TABLE I:** Baseline Characteristics of the Study Sample*

Characteristic	Responders to PROMs Questionnaires	Non-Responders to PROMs Questionnaires†	P Value‡
Total	66,769	25,167	—
Sex			
Male	29,040 (43.5%)	10,620 (42.2%)	
Female	37,729 (56.5%)	14,547 (57.8%)	<0.001
Age§ *(yr)*	69.74 ± 9.00	69.01 ± 10.2	<0.001
Area deprivation quintile#**			
Mean§	2.80 ± 1.35	2.97 ± 1.37	<0.001
1	14,328 (21.5%)	4,572 (18.2%)	
2	15,413 (23.1%)	5,399 (21.5%)	
3	14,720 (22.0%)	5,530 (22.0%)	
4	11,989 (18.0%)	4,905 (19.5%)	
5	9,465 (14.2%)	4,445 (17.7%)	
Living arrangements#			
Not living alone	48,092 (72.0%)	17,533 (69.7%)	
Living alone	16,692 (25.0%)	6,817 (27.1%)	<0.001
Preoperative/baseline OKS			
Mean§	18.62 ± 7.63	16.81 ± 7.76	<0.001
Median††	18 (13, 24)	16 (11, 22)	
Poor (<27)	57,977 (86.8%)	21,918 (87.1%)	
Fair (28 to 33)	6,660 (10.0%)	1,790 (7.1)	
Good (34 to 41)	2,031 (3.0%)	520 (2.1%)	
Excellent (42to 48)	101 (0.2%)	33 (0.1%)	
Duration of symptoms#			
Mean§	2.62 ± 1.00	2.71 ± 1.16	<0.001
≤5 yr	38,214 (57.2%)	13,815 (54.9%)	
>5 yr	28,136 (42.1%)	10,981 (43.6%)	
Comorbidities			
None reported	22,518 (33.7%)	8,124 (32.3%)	<0.001
1 or more	44,251 (66.3%)	17,043 (67.7%)	

* Data are presented as the number of patients, with the percentage in parentheses, unless otherwise noted.

† Baseline and/or 6-month follow-up knee score data were missing/incomplete.

‡ P values from Pearson chi-square test for comparison of proportions or t test for comparison of means.

§ Data are presented as the mean and the standard deviation.

# Missing <4% of data.

** 1st quintile is least deprived, 5th quintile is most deprived.

†† Data are presented as the median, with the interquartile range in parentheses.

In the study population, 56.5% of the patients were female, the mean age was 69.7 ± 9.0 years, and 25.0% of the patients lived alone. The mean preoperative knee score was 18.6 ± 7.6, and the majority of patients (86.8%) were classified as “poor” for pain and function. The average duration of symptoms prior to surgery was 2.6 years, and 66.3% of the patients reported ≥1 comorbidity (Table I).

The overall outcomes were good, with a mean improvement of 15.2 ± 9.9 in the knee score at 6 months postoperatively, which is 5 times the minimum clinically important difference. Only 10.8% of the patients failed to achieve the minimum clinically important difference. The mean knee score at 6 months postoperatively was 33.8 ± 10.0, with a quarter of the patients still classified as having a “poor” score. The distribution of knee scores preoperatively and postoperatively and the distribution of change in scores are illustrated in Figures 2-A, 2-B, and 2-C.

**Fig. 2-A:**
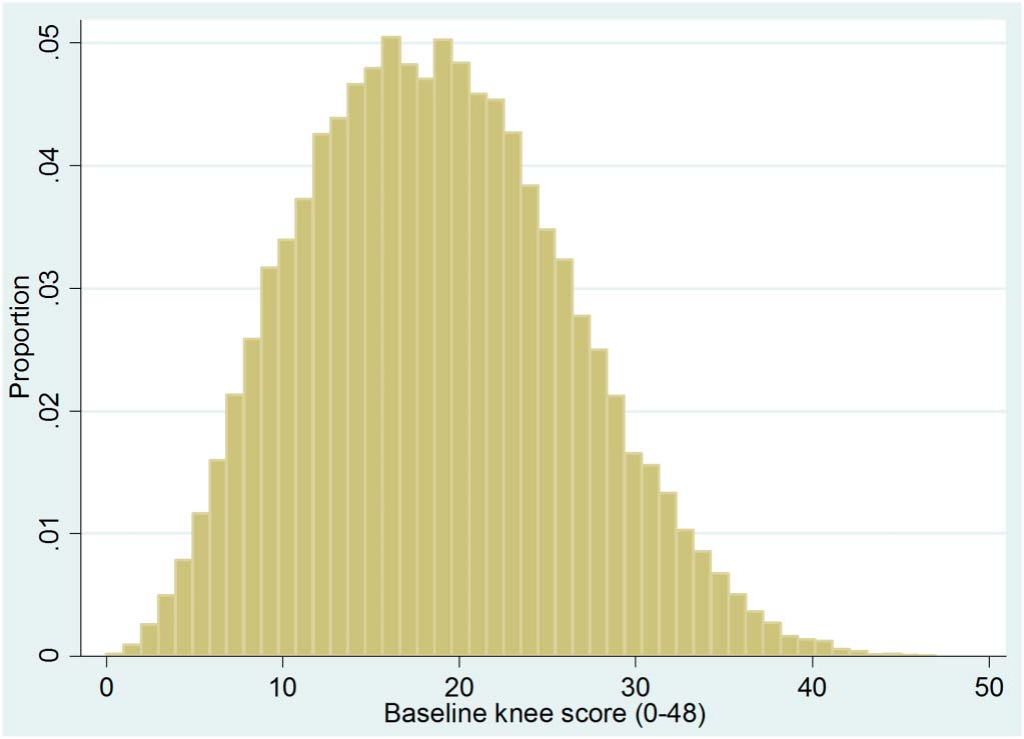
Graph showing the distribution of preoperative knee scores.

**Fig. 2-B:**
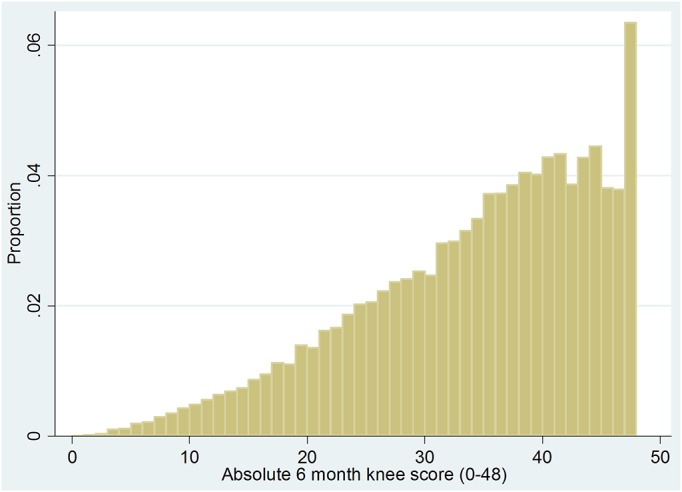
Graph showing the distribution of postoperative knee scores.

**Fig. 2-C:**
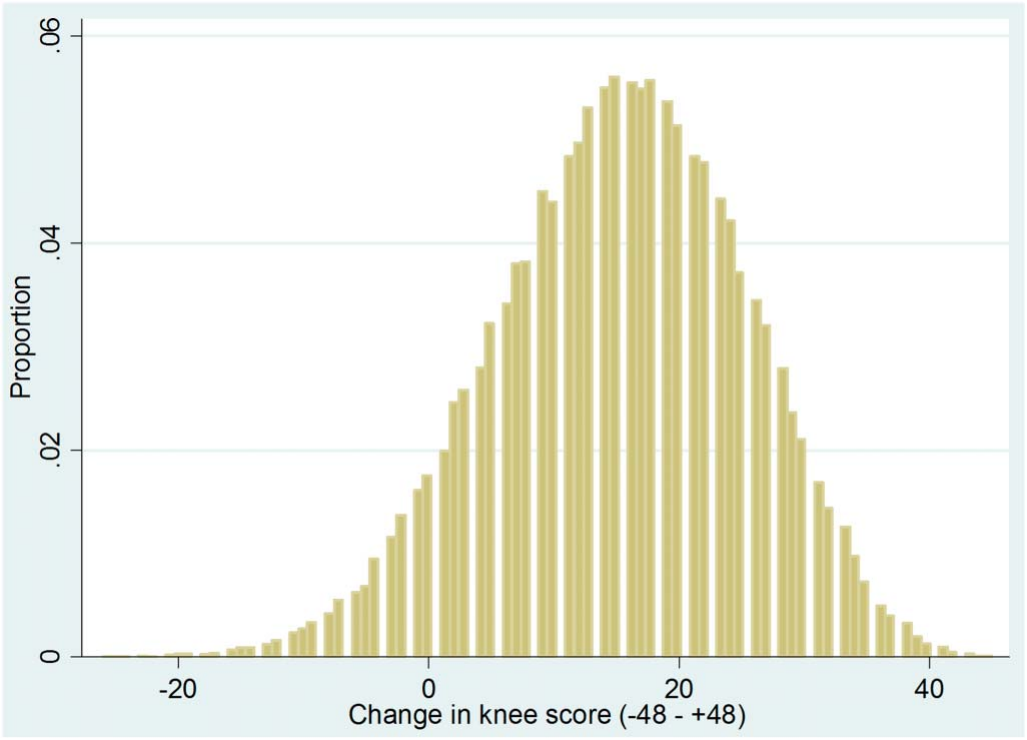
Graph showing the distribution of change in knee scores.

### Crude Results

Female patients, patients with greater area deprivation, and the youngest and oldest age groups had the worst preoperative knee scores (Table II). The magnitude in difference was approximately 3 points on the OKS.

**TABLE II:** Raw Knee Scores by Sociodemographic Group

Characteristic	No. of Patients	Raw Preoperative Score*	Raw 6-Month Postoperative Score*	Raw Change in Knee Score*
Sex				
Male	29,040	20.47 ± 7.73	34.89 ± 10.02	14.42 ± 10.08
Female	37,729	17.19 ± 7.24	33.03 ± 9.94	15.84 ± 9.83
Age				
≤50 yr	1,443	15.85 ± 7.23	29.67 ± 11.51	13.81 ± 11.14
51 to 65 yr	19,810	17.84 ± 7.51	32.73 ± 10.74	14.90 ± 10.27
66 to 75 yr	26,857	19.23 ± 7.56	34.70 ± 9.70	15.47 ± 9.83
76 to 85 yr	16,808	18.98 ± 7.74	34.19 ± 9.30	15.21 ± 9.69
≥86 yr	1,851	16.98 ± 7.68	33.33 ± 9.53	16.35 ± 9.69
Area deprivation quintile†‡				
1	14,328	20.14 ± 7.64	35.58 ± 9.11	15.44 ± 9.58
2	15,413	19.21 ± 7.66	34.83 ± 9.54	15.61 ± 9.75
3	14,720	18.63 ± 7.56	34.03 ± 9.93	15.40 ± 9.98
4	11,989	17.70 ± 7.46	32.67 ± 10.42	14.97 ± 10.23
5	9,465	16.45 ± 7.21	30.67 ± 10.78	14.22 ± 10.42
Living arrangements‡				
Not living alone	48,092	18.76 ± 7.62	34.01 ± 10.08	15.25 ± 9.97
Living alone	16,692	18.19 ± 7.62	33.36 ± 9.82	15.17 ± 9.90

* Data are presented as the mean and the standard deviation. The scoring system ranges from 0 (worst knee symptoms) to 48 (no symptoms).

† 1st quintile is least deprived, 5th quintile is most deprived.

‡ Missing <4% of data.

At 6 months postoperatively, all sociodemographic groups had improved, and male patients, patients 66 to 75 years of age, patients with the least area deprivation, and patients who did not live alone had the best outcomes.

Female patients, patients ≥86 years of age, and patients with the least area deprivation experienced the greatest change in symptoms postoperatively.

### Adjusted Results

The crude pattern remained after adjustment for confounding factors, with female patients, patients with greater area deprivation, and both the youngest and oldest age groups reporting the worst preoperative symptoms (Table III). At 6 months postoperatively, these sociodemographic differences were maintained.

**TABLE III:** Sociodemographic Variation in Preoperative and Postoperative Knee Scores and in Change in Knee Score: Adjusted Models*

Characteristic	Preop. Variation in Knee Scores†	P Value	6- Month Postop. Variation in Knee Scores‡	P Value	Partially Adjusted Difference in Knee Scores‡	P Value	Fully Adjusted Difference in Knee Scores§	P Value
Sex								
Male#	0		0		0		0	
Female	−3.30 (−3.42 to −3.18)	<0.001	−1.83 (−1.98 to −1.67)	<0.001	1.49 (1.33 to 1.65)	<0.001	−0.30 (−0.45 to −0.15)	<0.001
Age								
≤50 yr	−3.07 (−3.47 to −2.68)	<0.001	−4.87 (−5.39 to −4.34)	<0.001	−1.88 (−2.42 to −1.34)	<0.001	−3.51 (−4.00 to −3.02)	<0.001
51 to 65 yr	−1.19 (−1.32 to −1.05)	<0.001	−1.83 (−2.01 to −1.64)	<0.001	−0.65 (−0.83 to −0.46)	<0.001	−1.29 (−1.46 to −1.12)	<0.001
66 to 75 yr#	0		0		0		0	
76 to 85 yr	−0.24 (−0.39 to −0.10)	<0.001	−0.38 (−0.57 to −0.19)	<0.001	−0.15 (−0.35 to 0.05)	0.130	−0.27 (−0.46 to −0.09)	0.003
≥86 yr	−2.38 (−2.74 to −2.03)	<0.001	−1.40 (−1.87 to −0.92)	<0.001	0.99 (0.50 to 1.47)	<0.001	−0.30 (−0.74 to 0.14)	0.185
Area deprivation								
Increase per deprivation quintile	−0.80 (−0.84 to −0.76)	<0.001	−1.08 (−1.14 to −1.02)	<0.001	−0.28 (−0.33 to −0.22)	<0.001	−0.71 (−0.76 to −0.66)	<0.001
Living arrangements								
Not living alone#	0		0		0		0	
Living alone	0.36 (0.22 to 0.50)	<0.001	−0.09 (−0.27 to 0.10)	0.351	−0.48 (−0.66 to −0.29)	<0.001	−0.27 (−0.44 to −0.10)	0.002

* Data are presented as the differences in mean scores per group (with the 95% CI in parentheses), except for area deprivation, which shows the average change in score per quintile increase in area deprivation.

† Adjusted for all variables in the table, as well as for duration of knee symptoms and comorbidities.

‡ Adjusted for all variables in the table, as well as for the duration of symptoms, comorbidities, postoperative complications, knee implant type, and method of knee constraint.

§ Adjusted for all variables in the table, as well as for duration of symptoms, comorbidities, postoperative complications, knee implant type, method of knee constraint, and preoperative/baseline score.

# Reference group.

In our partially adjusted model for change in score, female patients improved more than male patients, and younger patients, patients with greater area deprivation, and patients who lived alone showed less improvement. However, when the differences in preoperative score were accounted for in the fully adjusted model, female patients showed fractionally less benefit than male patients, although the magnitude was clinically negligible. The adverse effects of a greater area deprivation and a young age at the time of surgery also increased after accounting for differences in preoperative score.

Although significant, the differences in improvement between most sociodemographic groups were of small magnitude. Age and area deprivation were the only factors with effects that approached a clinically noticeable difference. Cumulatively, sociodemographic factors explained <1% of the total variability in improvement in knee scores (0.50% for sex, 0.13% for age, 0.15% for area deprivation, and <0.01% for living arrangements). In contrast, preoperative knee scores explained 14% of the variability in benefit.

There was some statistical evidence of an interaction between age and sex (likelihood ratio test, p < 0.001) and between age and deprivation (likelihood ratio test, p < 0.001), such that the apparent disadvantage of younger age was greater in female patients than in male patients and the disadvantage of a greater area deprivation seemed to affect younger people more strongly than older people. However, the differences in effects were of a very small magnitude, so they are not presented here. Adjustment for BMI in the sensitivity analysis also made very little difference to model estimates and so is not shown.

We also explored whether sociodemographic factors were related to the risk of postoperative complications. Overall, the differences in risk were small. Females had a lower risk of complications compared with males (odds ratio [OR], 0.86; 95% CI, 0.83 to 0.89; p < 0.001), and those who lived alone had a fractionally increased risk of complications (OR, 1.06; 95% CI, 1.01 to 1.10; p = 0.007). Interestingly, patients ≤50 years old appeared to have a slightly increased risk of complications compared with the central age category of 66 to 75 years (OR, 1.13; 95% CI, 1.01 to 1.26; p = 0.033), and those from areas of higher deprivation had a fractionally lower risk of complications (OR, 0.97; 95% CI, 0.96 to 0.99; p < 0.001), although the magnitude of these differences was extremely small. These findings suggest that further research would be useful to identify whether the effect of sociodemographic factors varies for different types of complications.

## Discussion

We found that patients from more deprived areas, female patients, and younger patients reported worse symptoms prior to TKR. The same groups also had worse symptoms at 6 months postoperatively, suggesting that factors upstream of surgery may be the main drivers of variation in symptoms across the disease course. The majority of patients reported large improvements in symptoms. Adjusting for preoperative scores increased the negative effect of area deprivation on the change in OKS (from −0.28 to −0.71), whereas for female patients it reversed the direction of effect (from 1.49 to −0.30), highlighting the impact of accounting for preoperative scores. For example, if we compare patients from the best and worst deprivation quintiles, there is an almost 3-point difference in the improvement of the PROMs score. Although there is some controversy as to what change in the PROMs score represents the minimum clinically important difference, it has been suggested that it could be <3 or between 3 and 5^39^. The other differences in improvement were small, with the most noticeable disadvantage observed in patients <50 years old. However, overall, sociodemographic factors explained only a fraction of the total variability in improvement and are not a key factor in determining benefit from surgery.

Our results are consistent with those of previous studies that have shown that female patients^44-46^ and the deprived^44,45^ have less provision of TKR relative to need, suggesting that clinicians and/or patients have a higher threshold for surgery for these groups. Younger patients may be considered “too young”^47^ and may wait for worse symptoms before considering surgery. Other studies from the United Kingdom^5,27^ have shown that greater area deprivation predicted slightly worse outcomes, with comparable estimates of effect. Other studies^20,21,23,24,27,46,48,49^ also have shown that male and female patients have comparable outcomes or that female patients do slightly worse than male patients. The small sex-related differences that were observed in the present study suggest that smaller studies may have been underpowered to detect differences. Our results are broadly in line with the findings from a 2012 study from the U.K.^27^ that showed that older patients had fractionally worse outcomes at 6 months postoperatively. However, we make the separate qualifying point that the benefit of surgery for older patients is comparable with or better than that for younger patients after accounting for preoperative scores. An Oxford study^28^ demonstrated that the youngest patient group seemed to experience fractionally more benefit compared with older patients, which contradicts our observation; however, the data in that study were not adjusted for confounding factors that were likely to have an important effect on results (e.g., older patients people tend to experience more comorbidities, which may partly explain the worse outcome observed in older patients).

A possible explanation for the effects of age and sex on outcomes is that female patients and patients who develop osteoarthritis at a younger age have a more severe or faster-progressing disorder^50^ that may be less responsive to treatment. This notion is supported by the finding that a shorter duration of symptoms was associated with an increased risk of not achieving a minimum clinically important difference (data not shown). Differing expectations regarding surgery also could have a role in the finding that younger patients seem to benefit less. However, this effect is likely to be small as only a few of the OKS questions are subjective. The slight adverse effect of living alone could be explained by lower social support, loneliness, lower confidence in resuming activities, and less mobilization following surgery^51,52^. Alternatively, it may be that those who live alone need to cope with everyday living demands unaided and are in fact active prematurely, thereby compromising their recovery. Additional research on the mechanism for the effect of area deprivation on outcome could be useful. For example, it may be that more deprived areas tend to have weaker surgical teams because of issues with recruitment, turnover, or inexperienced surgeons, factors that may be associated with poorer outcomes.

### Strengths and Limitations

To our knowledge, this is the largest study exploring the effect of sociodemographic factors on the outcomes of TKR. This study involves data from a national population-based joint registry, combined with independently and prospectively collected patient-reported outcome data. A range of key surgical and clinical confounders (including symptom duration, comorbidities, knee implant type, knee constraint type, postoperative complications, and demographic factors of interest) were controlled for. The study was highly powered to detect differences in outcomes.

Our analyses were restricted to Caucasian patients, which limits the generalizability of our findings. It is unclear whether the associations that we found with age, sex, area deprivation, and living arrangements would be the same within other ethnic groups or whether there may be interactions between ethnicity and the other variables. Another limitation was the lack of information on other potentially confounding factors such as psychological well-being, smoking, diet, compliance with rehabilitation exercises, mobilization, and use of painkillers^27,53-55^. Smoking and obesity are believed to have an adverse effect because they are associated with a higher rate of postoperative complications ^56,57^. We adjusted for postoperative complications, so we expect that the residual effect of smoking and obesity should be minimal. The sensitivity analysis on BMI provides some evidence that the residual effect of obesity is minimal because this analysis showed little effect on the results of additional adjustment for BMI. However, because BMI was recorded at the time of the operation, this information was most likely missing at random and is therefore unlikely to have introduced bias in the results. Other work by our group to impute missing BMI data has shown that BMI does not make a difference in outcomes^58^. We did not adjust for surgeon effects, which could confound the findings if high-volume surgeons have better outcomes. However, the U.K. health-care system makes differential access to such surgeons unlikely, and we suspect that, because these surgeons probably operate on more difficult cases, adjusting for surgeon experience actually would make the observed differences even wider.

Approximately one-quarter of patients gave insufficiently complete responses for their change in knee score to be calculated, a finding similar to that reported in other studies^5,42,57,59^. Non-responders tended to come from more deprived areas and to have fractionally worse health before surgery, making a non-response selection bias possible. Non-responders may have disengaged with services because of disappointing outcomes, which could have resulted in underestimation of the area deprivation effect.

Over a quarter of NJR records could not be sufficiently linked to either HES or PROMs datasets. The characteristics of the unlinkable records were not available so it is hard to assess the impact of these missing records. Most were because of omissions in routine HES data, making it plausible that these records were missing at random; these omissions are likely to have led to underestimation of associations. In the context of the small effect sizes found, further exploration of the missing data (if possible) would be useful.

One important implication of our findings is that there is no basis for “age rationing” of TKR. Elderly individuals can sometimes be considered “too old” to benefit from surgery or less likely to benefit when compared with younger people^60,61^. This may be a perception of both general practitioners and elderly patients themselves, who may avoid surgery for altruistic reasons or who may not appreciate the benefits of surgery^47^.

### Overview

In conclusion, the majority of patients achieved substantial improvement in symptoms following TKR. Patients <50 years old and those from deprived areas benefited less from surgery and achieved worse absolute scores at 6 months postoperatively. Female patients had worse preoperative and postoperative symptom scores than male patients but benefited comparably from surgery. Despite some differences, overall sociodemographic factors were not strong predictors of the benefits of surgery. As sociodemographic factors are associated with the threshold at which surgery is performed, future work should perhaps focus on factors upstream of surgery that may be life-course determinants of osteoarthritis and the drivers of variation in knee pain and function across the course of disease.

## Appendix

A table showing the OKS questions from the PROMs questionnaire is available with the online version of this article as a data supplement at jbjs.org (http://links.lww.com/JBJSOA/A42).
